# Understanding sexual and reproductive health needs of young women living in Zika affected regions: a qualitative study in northeastern Brazil

**DOI:** 10.1186/s12978-020-0869-4

**Published:** 2020-02-06

**Authors:** Debora Diniz, Moazzam Ali, Ilana Ambrogi, Luciana Brito

**Affiliations:** 1 Rights and Justice Regional Deputy Director - International Planned Parenthood Federation/Western Hemisphere Region (IPPF/WHR), Caixa Postal 8011, Brasília, DF 70094-971 Brazil; 2 Department of Reproductive Health and Research (RHR), World Health Organization, Avenue Appia 20, CH-1211 Geneva 27, Switzerland; 3 Researcher at Anis - Institute of Bioethics, Caixa Postal 8011, Brasília, DF 70094-971 Brazil; 4 Director of Research at Anis - Institute of Bioethics, Caixa Postal 8011, Brasília, DF 70094-971 Brazil

**Keywords:** Zika virus, Sexual and reproductive health, Public health, Young women, Brazil

## Abstract

**Background:**

In 2016, the World Health Organization declared a Public Health Emergency of International Concern due to Zika’s association with microcephaly and other neurological disorders. Brazil was the epicenter of this epidemic and the most affected region has the lowest Human Development Index and the highest rates of adolescent pregnancy. Despite the end of the epidemic, Brazil continues to be the epicenter of Zika illness. This study examined the barriers faced by young women who seek sexual and reproductive health (SRH) care services living in affected areas and their attitudes towards SRH needs and the available services.

**Methods:**

Individual semi-structured interviews were conducted with 22 young women, aged 14–24 years in three Zika affected municipalities in the Brazilian Northeast. This qualitative research used thematic analysis for data analysis.

**Results:**

Almost half (*n* = 10) of the participants had their first pregnancy during adolescence (from 12 to 19), all of which were unintended. Lack of information and barriers to access family planning were found to contribute to the unmet need for contraception. Participants reported knowledge gaps about contraception. Zika was not considered a health concern and participants were unaware of the possibility of Zika’s sexual transmission.

**Conclusions:**

The young women’s knowledge and attitudes towards their SRH needs highlight the barriers to access care. It also implies that comprehensive, biopsychosocial and political, understanding is necessary in order to adequately provide SRH to this population and meet their needs. The government should place women at the center of any public health response to an emergency affecting women of reproductive age and focus on improving access to information and family planning services in a culturally and age appropriate manner.

## Plain English summary

Congenital Zika Syndrome is a novel syndrome due to in-utero Zika virus infection. It was discovered after Brazil had become the epicenter of the Zika epidemic in 2016. The mosquito born Zika virus has been shown to cause microcephaly and other severe abnormalities in the developing fetus and child. It can also affect the pregnancy. Due to its severe effects, the World health Organization declared a Public Health Emergency of International Concern in 2016 and efforts were made to learn more about the consequences of this infection. It has been found that the Zika virus is also transmitted sexually and that the most affected are young, poor, underserved afro and native Brazilian women.

This study investigates the sexual and reproductive health (SRH) needs of young women living in Zika affected areas. Women interviewed were seeking SRH services at three different northeastern cities’ public health services between November 2017 and July 2018. Ten out of 22 had their first pregnancy while still in adolescence; all of these pregnancies were unintended. Zika was not a health concern and they were not aware that Zika could be sexually transmitted. Lack of information and barriers to access aggravated their unmet SRH needs.

In order to improve SRH of women at most risk, they need to be placed at the center of any public health response to an emergency. It is only by understanding their needs and the obstacles they face when seeking care that these issues will be adequately addressed.

## Background

Zika virus is a mosquito borne virus that has been shown to cause microcephaly and other severe brain anomalies in newborns when the mother is infected prenatally [1, 2]. The Northeast of Brazil was the epicenter of the Zika epidemic; and for the fifth time in history the World Health Organization (WHO) declared, on February 1st, 2016, a Public Health Emergency of International Concern (PHEIC) due to the association of Zika with microcephaly and other neurological disorders. On November 18th of that year, the PHEIC was lifted [3]. The Brazilian Ministry of Health also encouraged women, planning to postpone pregnancies, to seek contraceptive methods at Basic Health Units [4]. This underserved Northeast Brazilian geographic region has the highest adolescent pregnancy rates, lowest levels of formal schooling and scarce access to health services [5, 6]. This region has several social-economic challenges, where most of the population does not have access to basic services such as running water, access to health and information and where the mosquito vector *Aedes aegypti* and tropical diseases like dengue have been part of everyday life for generations [6]. All these factors influence the health of the population and make it vulnerable to outbreaks and other health related challenges.

Given the consequences of Zika infection on pregnancy and fetal development, it has been understood as an enduring public health task. However, most of the efforts in response to the epidemic were related to vector control, development of adequate methods for diagnosis and the possibility of a vaccine. Little attention was given to the social impacts of Zika virus and how to provide information to a population with low literacy and little access to health services [7].

There is a general lack of information about sexual and reproductive health (SRH) and there are barriers to access public health facilities for family planning. The Brazilian Public Health System (SUS) offers several contraceptive methods for free [8]. Although studies show that Brazil has a relatively high modern contraceptive prevalence, there is low use of long-acting reversible contraceptives (LARCs) in the public sector, and inequities exists [9, 10]. In 2010, around 50–55% of all births in Brazil resulted from unintended pregnancies [11]. It is estimated that in Brazil, one in five children are born to adolescents between the ages of 10 and 19 years old [12–14]. Despite the observed decline in live births in Brazil since the emergence of the epidemic, there were no changes in the health policies and no evidence that there was an increase in demand for long-term contraceptive methods [12, 14]. These findings demand attention and evaluation in order to develop evidence-based guidance for strengthening health systems and to prevent harmful practices.

The Zika outbreak has exposed how access to sexual and reproductive health and its needs are a major public health issue, but there is a dearth of social and qualitative research. This paper aims to describe the young women’s perceptions and their needs for sexual and reproductive health services as well as the barriers they face when seeking sexual health and reproductive planning in Brazilian public health care facilities located in regions most affected by the Zika virus. Although our study might not represent the entire population’s view on the matter, it highlights the voices of the women most at risk of the negative impacts of the outbreak.

## Methods

### Design

This qualitative research used the thematic analysis method. Our aim was to investigate in a purposive sample and through in-depth analysis a conceptual description of young women’s sexual and reproductive health needs as well as the barriers they faced trying to access health care services. Because of that, this study did not intend to specify frequencies, nor produce generalizing statistical inferences.

### Recruitment of participants

The participants of this qualitative study were young women seeking sexual and reproductive health services in the Brazilian public health facilities of three Zika affected municipalities in the Northeast of Brazil: São Luís and Balsas in Maranhão state, and Santana do Ipanema in Alagoas state. Participants’ ages ranged between 14 and 24 years old. This study will refer to its the participants which includes adolescents (aged 10–19) and youths (aged 15–24), as young women. The study setting was selected based on the three Brazilian health care facilities place-based Sentinel Sites Evaluation (SSE) activities established by the research study sites WHO Human Reproduction Program (HRP). Health care workers invited women seeking sexual and reproductive health services at these sites between November 2017 and July 2018 to participate in the study. An informed opt-in procedure was used, participants were only contacted by the research team after providing their consent for the contact to the health care workers. Data was stratified by age and only women younger than 24 years old were considered for this analysis.

### Procedure and data analysis

Women researchers performed individual, in-depth interviews with 22 participants. The interviews lasted from 40 to 90 min. They were semi-structured interviews and followed a topic guide, but it took place as a conversation in which the researchers promoted a safe, comfortable environment to enable a comprehensive and candid record. Initial questions were on sociodemographic data, such as age, color, ethnicity, occupation, level of education, marital status, number of children, religion if any, and address. Questions about their attitude and knowledge on Zika virus, CZS, sexual transmission of Zika, contraception, abortion, pregnancy, and SRH needs and services, followed. The participants were invited to choose the places of the interviews, most of them preferred their homes (*n* = 20).

﻿ After each interview was transcribed, microanalysis was used as the analysis strategy. Microanalysis is a type of detailed analysis of data in which line by line or sentence by sentence of documents are examined at the beginning of the study [15]. With an inductive approach to the analysis initial codes were generated. Codes were collated and those with similar pattern of meaning were grouped in order to identify themes [15, 16]. The coding process was used in order to focus the data analysis on what emerged from the empirical evidence. The coding process was systematically discussed between the researchers and was initiated after each interview was transcribed in order to identify core information during the data collection phase. Transcribed interviews collected by two researchers were subsequently coded accordingly [15, 16]. Coded interviews and the data were tabulated in order to identify the patterns and review themes. Standards of coding were compared, and, in case of discrepancy, the coding method was discussed by the research team. In this analysis two themes emerged: lack of information and barriers of access to reproductive and sexual health services.

This study protocol was approved by the University of Brasilia Research Ethics Committee for Humanities and Social Sciences – CAAE: 66502017.0.0000.5540 and WHO Ethics Review Committee. All the participants provided informed consent in order to participate in the study in accordance with Brazilian regulations which guarantee autonomy and confidentiality about the sexual and reproductive health decisions and practices of the individual.

## Results and discussion

### Participant characteristics

Participants in the three municipalities had similar characteristics. The majority of them declared themselves as Afro-Brazilians (*n* = 18). Of the participants who declared having a religion (*n* = 17), Catholicism (*n* = 10) and Evangelicalism (*n* = 7) were reported. Only one participant said she had no religion. Eight participants had a very low level of formal schooling: they stopped their studies while in elementary school, a higher proportion than expected for this age group. According to the Brazilian National Household Survey, PNAD 2015, the national rate of adolescents between 17 and 19 years old with incomplete elementary education was less than 15% [6]. Among participants with very low formal schooling, only one had not experienced an adolescent pregnancy. Twelve participants completed high school or discontinued their studies during high school. Two participants were currently attending undergraduate courses.

Of the 22 participants, 12 had children. Ten participants had their first delivery during adolescence (from 12 to 19 years old) and all of the participants who had a childbirth experienced unintended pregnancy. Participants who had a low level of formal schooling reported that pregnancy was a relevant factor for leaving school – as a 19-year-old participant who had her first child at 14 years old reported: “*When I least expected it, I got pregnant. Then I had to accept it, right? I had to leave school. Now I have two children. I’m trying to look for a job here, but it’s difficult because I do not have schooling.*” Studies reveal that adolescent pregnancy could be considered a relevant factor for discontinuity of school. In this sense, improving adolescents’ SRH is essential to their social and economic well-being [17, 18]. Access to information and adolescent-friendly health services are key factors to reduce unintended early pregnancies [19–21].

Generally, women are considered to have an unmet need for contraception if they are fertile or are sexually active and want to delay or avoid becoming pregnant but are not using a contraceptive method [22]. However, in this study it is understood that an unmet need could also be expressed as lack of information about SRH and barriers to access these kind of health services. The lack of information unfolds in two main aspects: knowledge gaps about contraceptives use; and misconceptions on contraceptive methods adverse effects and their effectiveness. Communications failure and unawareness about Zika and its consequences were barriers women faced when seeking SRH in high risk areas.

### The information gap on sexual and reproductive health care

#### Lack of knowledge on contraceptive use

Despite the increase in use of contraceptives in Brazil [23, 24], the young women were unaware of how to use them effectively – or even how to access these contraceptive methods. They reported having little trust in short acting contraceptive methods such as oral and injectable contraceptives – which are commonly offered by the Brazilian public health services and have the highest usage prevalence in Brazil [25]. The young women justified their mistrust correlating two experiences: their personal unintended pregnancies experience and the contraceptive method used at that moment, as explained by a 16-year-old participant: “*We use condoms, but we don’t know the right way to use them. That is why it happens that people get pregnant when they use condoms, right? That is what happened with me.*”

Some young women reported that they were not using any contraceptive method, as described by an 18-year-old adolescent who experienced a repeat pregnancy within one year of the birth of her first child. Her first delivery was when she was 16 years old: “*I wasn’t taking anything to avoid it. I didn’t know it was possible to get pregnant so fast.*” This finding is indicative of missed opportunities to inform adolescents about family planning, be it at school and/or during previous prenatal and postpartum care. Studies suggest that adolescents who are not actively seeking to prevent pregnancy are predisposed to it [26–28]. Population studies in Brazil indicate a large proportion of unplanned or unwanted pregnancies [5, 29–31]. Data from the National Demographic and Health Survey (PNDS) from 2016 revealed that 19% of women in Brazil experienced an unwanted pregnancy on the day of the interview; in the Northeast of the country – the region where the study was conducted – the proportion reached 22,5%. In Brazil, more than half of all births were reported as unwanted or mistimed [11, 24, 29].

The risk of an unintended pregnancy during contraceptive method changes was also not stressed and discussed with the women at risk. A 24-year-old woman who had her first delivery at 17, the second at 20 and the third delivery at 23 years old, described her experience at the family planning facility: “*They told me to use the IUD after the postpartum period. But I was only able to get out of the house after 40 days. Then I got there, and they said I could not have it inserted because more than 30 days had passed. That I would have to wait for my period to come.*” Lack of proper and up to date information about SRH, in particular, contraceptive methods use, and the risk of an unintended pregnancy leads to inadequate compliance thus adding to the dissatisfaction with family planning services.

One of the study participants did not know about the Zika virus’ potential health effects or the risks of congenital syndrome. Mosquitos were described by most of them as the main vector of transmission of the Zika’s illness and microcephaly in babies as one of the consequences of the virus. However, none of the young women knew about Zika’s sexual transmission, or the recommendation for condom use during the pregnancy. This finding is significant for an area that was affected by the Zika epidemic and continues to be at risk for the illness.

#### Misconceptions on contraceptive methods adverse effects and effectiveness

Shared community perception about contraceptive methods matters in promoting its acceptance or refusal. The young women expressed concerns about the only LARC available to them in the Brazilian public health system, the copper intrauterine device (IUD). Women who fear IUD insertion justify their fright based on stories heard in their communities. Most common stories are about women who have had chronic diseases such as cancer, or women who become pregnant after the device’s placement. They also explain the fear of inserting a foreign object into their bodies and about the copper decomposing inside their womb. Other misconceptions were also related as demonstrated by the literature on the supposed side effects of the IUD such as abortion, pelvic inflammatory disease, infertility or pain [32].

At the same time, the copper IUD was considered by the young women a good contraceptive choice when they heard the experiences of other women of their community. The experience of other women with whom they have interpersonal ties, close and trusting relationships is considered an important aspect before deciding whether to use the IUD. Most of those who chose the IUD sought the service after the indication of another woman who was a friend or a family member (*n* = 15). A 17-year-old adolescent described why she decided to use the LARC: “*It was my friend who told me about the IUD and I like it. After I had my daughter, I’ve been thinking that I’m pregnant, all the time. So, I chose the IUD in order to make sure I’m not pregnant.*” Understanding the influence of the close community in young women’s reproductive choices should be appreciated by the public health system and providers.

Female sterilization was understood by many young women as the only truly effective method, as described by this 16-year-old participant who became pregnant at the age of 15: “*Look, I’m even afraid to have sex, I’m afraid! When I remember the amount of work this one gives me, and she is still a baby. I wanted the (tubal) ligation, but I’m too young. That’s why I’m going to get the IUD and when I turn 24, I’m going to ask to get the sterilization.*” One possible way to understand this perception towards the irreversible contraceptive method could be due to reproductive coercion as well as stereotyping of women in health care. The category of reproductive coercion could be influencing young women’s understanding of female sterilization [33, 34]. It suggests, nonetheless, that gaps in young women’s knowledge about birth control have important consequences in their fully informed choices about contraceptive methods.

The young women reported suspending the continued use of hormonal contraceptive methods due to unwanted side effects on their body or health. They also described that the misconceptions regarding the utilization of hormonal methods were related to some health care professionals who asked women to stop using pills or hormonal injections in order to continue the menstrual cycle to “*cleanse the body.*” The young women also related that some health care providers recommended that the women stop using methods in order to switch them or simply to wait a period of time before starting a new contraceptive method. These professionals, however, did not provide any evidence-based explanations or offer an alternative method of birth control. Studies demonstrated that Brazilian women – mainly among those with less years of schooling and low socioeconomic status – view menstrual bleeding as a sign of health, fertility, and of not being pregnant [35]. However, it is not yet fully understood how the women’s preference for monthly menstruation can affect their sexual and reproductive health.

### Barriers to access sexual and reproductive health care

#### Challenges in overcoming communication failures

Planning when and if the young women want to experience pregnancy is an important issue, but they faced barriers to access SRH services. The wish to postpone pregnancy was highlighted by all young women who had experienced childbearing. Most of those who have a child (*n* = 10), sought medical advice to start a contraceptive method, only after the first childbirth. These young women, especially those with little schooling, reported experiences in which there was little understanding of the information offered by the health care professional. A 15-year-old adolescent explained the barrier she faced: “*The doctor and the nurse speak in a very complicated manner, they speak things that we cannot understand. But there is also embarrassment to ask about the same thing again.*”

Young women reported their appreciation of discussion groups and informative lectures on available contraceptive methods and SRH. They specifically identified the utilization of visual aids, such as anatomical models, and handling of samples as particularly helpful for providing information as well as for establishing rapport with health care providers. Regarding their queries about the IUD, young women affirmed that a detailed presentation about the method, with step-by-step demonstrations of its insertion in a pelvic model would be an important means for providing information and addressing their concerns.

Communication barriers are one of the observed difficulties these women face when trying to access SRH in these services. Adolescent girls declared that they usually do not seek the health care facilities for family planning services. Communication failure leads to the distancing between health services and the population. One of the reasons for the high unmet contraceptive need is the inability to deliver proper information to these women and girls in a manner that is respectful and also makes sense to them.

The persistent inadequate and inefficient communication between the health care facilities and the women contributes to their perception and attitudes about the services. In addition, the community perception of a long-standing state of deficits in the public health services available lead women to view them as unreliable. The long waiting periods for appointments, lack of desired birth control methods, such as LARCs, or the communication and information gaps were reported by women as circumstances that led them to not trust that the health care services would cater to their needs. In order to overcome this barrier to access, their preemptive attitude is to first seek SRH elsewhere, such as in their close community of friends and relatives and/or with a local pharmacist.

#### Unawareness and misinformation on Zika

Young women reported that Zika is no longer discussed during prenatal care. Mosquito repellents are provided at the public health services to pregnant women who are beneficiaries of a governmental income transfer program for very poor families called “Bolsa familia” [6, 36]. However, the recommended use of the mosquito repellent is usually not mentioned by the health providers during the visits nor any explanation is given as to why they should use it.

Zika is also no longer considered an infection that can affect their health or the development of their newborn and fetus. Zika infection was not a concern during the pregnancy of young women who experienced childbirth in late 2017 or 2018 (*n* = 7). Only young women who were pregnant during the epidemic in 2015 and 2016 (*n* = 6) reported being worried about the potential Zika effects of vertical transmission on their fetus’ health, as described by this 21-year-old participant: “*I got pregnant when the cases of babies with microcephaly were on TV. I was very afraid of Zika. Afraid that Zika would pass on to her.*” This worry ended once the infant was born with normal head circumference. Microcephaly was the only feature recognized as a consequence of Zika infection in the newborn.

These findings indicate a lack of health awareness regarding Zika virus and its effects. Zika is not perceived as a current issue that can affect women’s wellbeing. Participants refute the idea that after the end of the outbreak, Zika illness would still be a threat to their pregnancy and newborns. They justified their low level of concern by the fact that Zika is no longer broadcast on radio and television, is not part of the conversations in social gatherings, and is not even brought up by health professionals.

Although the number of Congenital Zika Syndrome (CZS) cases have decreased, cases continue to be reported. In 2018, 8680 probable cases of Zika virus disease were registered in Brazil, with an incidence rate of 4.2 cases/100,000 inhabitants [37]. According to the March 2019 bulletin from the Ministry of Health, 1657 suspected cases were reported in 2018 as CZS, 227 children have been confirmed as Zika-affected cases, and 839 are still under investigation [38]. Brazilian women continue to live with the main transmitting mosquito, *Aedes aegypti*. Social and environmental conditions of these women remain unchanged and Zika illness is still present in the country, especially in the Northeastern regions.

Zika in Brazil should not only be considered in epidemiological terms, but also as a picture of social and gender inequalities which increase the vulnerability of women and families most at risk [6, 7, 39]. Women, especially adolescents, affected by Zika in Brazil represent one of the most vulnerable groups in the country; in addition, they have a late start to prenatal care and live in areas where inequities and extreme poverty persist [6]. Lack of information and barriers to access SRH services reported in this study can serve as an urgent next step for prioritized public health policies.

## Methodological considerations

This study contributed to a deeper understanding of how young women’s attitudes towards their SRH needs exposes the barriers to access care. Two researchers did the interview transcriptions’ coding process separately and independently in order to increase trustworthiness of data analysis. In the few occasions of discrepancies during the data coding process, a third researcher who is experienced in the methodology used, analyzed the same data and discussed it with the research team in order to ensure its credibility and consistency of coding. Our study used thematic analysis to generate a conceptually informed interpretation of data thus increasing conformability and allowing for the creation of a conceptual description of women’s attitudes when seeking SRH in these locations during the period studied.

Our study is limited by the design and sampling selection. Women who did not access the SRH service were not part of this study, so conclusions regarding barriers to access may be underemphasized. Generalizability is limited due to the nature of the study design and further research is needed in order to better understand the sexual and reproductive services practices as well as unmet contraceptive needs in different contexts.

## Conclusions

Our data suggest that young women in Zika affected areas have knowledge gaps of Zika related illnesses and about SRH. Young women perceived that at the national level and also at the health care facility level family planning and Zika illness are no longer health concerns. The description of the young women’s perceptions and challenges provides a comprehensive overview of their SRH needs and barriers to access. Lack of information on contraceptive use and its effects along with communication failures regarding SRH and unawareness about Zika create access barriers and likely contribute to the unmet SRH needs of young women living in high risk areas for Zika.

The findings presented in this paper could contribute to developing guidance on health-care services in order to enhance better provision of SRH counseling and services to young women and adolescents living in Zika affected regions. To conclude, in order to provide culturally sensitive, age appropriate and effective SRH care for young women, it is essential that these young women and girls be placed at the center of any public health response to an emergency affecting women of reproductive age.

## Data Availability

The datasets generated during and/or analyzed during the current study are not publicly available due to ethical protection according to the International Ethical Guidelines for Health-related Research Involving Humans in order to guarantee autonomy, privacy and confidentiality regarding sensitive sexual and reproductive health matters investigated in this research, but are available from the corresponding author on reasonable request.

## Abbreviations

CZS: Congenital Zika Syndrome
HRP: Human Reproduction Program
IUD: Intrauterine Device
LARC: Long-acting reversible contraceptive
PHEIC: Public Health Emergency of International Concern
PNAD: Brazilian National Household Survey (in Portuguese: Pesquisa Nacional por Amostra de Domicílios)
PNDS: National Demographic and Health Survey (in Portuguese: Pesquisa Nacional de Demografia e Saúde)
SRH: Sexual and Reproductive Health
SSE: Sentinel Sites Evaluation
SUS: Brazilian Public Health System (in Portuguese: Sistema Único de Saúde)
WHO: World Health Organization
